# Numerical Simulation and Experimental Investigation of the Viscoelastic Heating Mechanism in Ultrasonic Plasticizing of Amorphous Polymers for Micro Injection Molding

**DOI:** 10.3390/polym8050199

**Published:** 2016-05-17

**Authors:** Bingyan Jiang, Huajian Peng, Wangqing Wu, Yunlong Jia, Yingping Zhang

**Affiliations:** 1 State Key Laboratory of High Performance Complex Manufacturing, Central South University, Changsha 410083, China; jby@csu.edu.cn (B.J.); huajian_peng@163.com (H.P.); yunlong.jia@tuhh.de (Y.J.); zyp57783@163.com (Y.Z.); 2 Institute of Polymer Composites, Hamburg University of Technology, Denickestrasse 15, Hamburg D-21073, Germany

**Keywords:** micro injection molding, ultrasonic plasticizing, viscoelastic heating, numerical simulation

## Abstract

Ultrasonic plasticizing of polymers for micro-injection molding has been proposed and studied for its unique potential in materials and energy-saving. In our previous work, we have demonstrated the characteristics of the interfacial friction heating mechanism in ultrasonic plasticizing of polymer granulates. In this paper, the other important heating mechanism in ultrasonic plasticizing, *i.e.*, viscoelastic heating for amorphous polymer, was studied by both theoretical modeling and experimentation. The influence mechanism of several parameters, such as the initial temperature of the polymer, the ultrasonic frequency, and the ultrasonic amplitude, was investigated. The results from both numerical simulation and experimentation indicate that the heat generation rate of viscoelastic heating can be significantly influenced by the initial temperature of polymer. The glass transition temperature was found to be a significant shifting point in viscoelastic heating. The heat generation rate is relatively low at the beginning and can have a steep increase after reaching glass transition temperature. In comparison with the ultrasonic frequency, the ultrasonic amplitude has much greater influence on the heat generation rate. In light of the quantitative difference in the viscoelastic heating rate, the limitation of the numerical simulation was discussed in the aspect of the assumptions and the applied mathematical models.

## 1. Introduction

Polymer plasticizing with ultrasonic energy and directly injection molding with ultrasonic sonotrode has been proposed especially for micro-sized parts in fields like electronics, medicine, or biotechnology [1,2,3,4,5]. The method is also called ultrasonic micro-molding, which has been developed for its unique potential in materials and energy saving [6,7]. In comparison with traditional micro injection molding, over 90% of the polymer can be saved by plasticizing just the small amount of polymer just as required for micro-sized parts [6]. This could be a significant cost factor for micro-molded parts, especially in the case of engineering polymers used for high-performance applications. Another advantage of ultrasonic micro-molding is that external heating for the plasticizing chamber and the mold could be spared due to the propagation of the energetic ultrasound in the molten material [6].

Despite some advantages over conventional micro-injection molding, the potential applications of ultrasonic micro-molding are still limited [5,6,7]. This could be related to the complexity of the ultrasonic micro-molding process which is still under development. One of the challenges of ultrasonic micro-molding process that has been confronted has been the plasticizing of just a small amount polymer as required. The plasticizing quality of the molten material could be very sensitive under concentrated ultrasonic energy in a very short time. Uneven plasticization and even polymer degradation have occurred very often due to improper handling [5,6,7]. To achieve controllable ultrasonic micro-molding, the characteristics and mechanisms of ultrasonic plasticizing have to be understood.

According to Michaeli *et al.* [1,2,3,4] there are two types of heat generation mechanisms, *i.e.*, the interfacial friction heating and the viscoelastic heating in ultrasonic plasticizing process. In our previous work [5], the interfacial friction heating of polymer granulates has been studied by numerical simulation and experimentation. It was found that the polymer interfacial heating occurs and lasts only until the interfaces disappear, meaning that it acts only at the initial stage of ultrasonic the plasticizing process. In other words, the viscoelastic heating might be the predominant heat generation mechanism for ultrasonic polymer plasticizing. However, as a new concept for polymer plasticizing in micro-molding, the mechanism that can clearly explain the viscoelastic heating effect has been rarely reported. On the contrary, extensive modeling efforts have been made to simulate the ultrasonic polymer welding process [8,9,10,11,12,13,14]. Both interfacial friction and volumetric viscoelastic heating were taken into consideration in the modeling. It was confirmed that the temperature increase at the welding interface can be attributed to the combined action of interfacial friction and viscoelastic heating and that the viscoelastic heating effect plays a major role in ultrasonic polymer welding.

Unlike the heat generated in ultrasonic polymer welding which is mainly confined to the interface area, the heat generated in ultrasonic polymer plasticizing should be released and transferred to melt the polymer in the whole chamber. This could be a significant difference for process modelling. In addition, modelling efforts were mainly concentrated on the temperature distribution in ultrasonic polymer welding for different materials and joint designs [10]. For the quantitative characterization of the viscoelastic heating and the influence mechanism of the process parameters, however, there has been only a very few literature report for the case of both ultrasonic welding [10,13,14] and ultrasonic plasticizing [5]. Therefore, the objective of this work is to study the characteristics and mechanisms of viscoelastic heating in ultrasonic plasticizing by both theoretical modeling and experimentation. The viscoelastic heating phenomenon was modeled on the basis of several models including the generalized Maxwell model, the Arrhenius and semi-empirical WLF models [11,12,15,16], which was solved numerically by the commercial FEM software ANSYS. The heat generation rate was calculated and measured quantitatively. The influence mechanism of the initial temperature of the polymer, the ultrasonic amplitude, and the ultrasonic frequency on the heat generation rate were studied.

## 2. Theoretical Modeling

### 2.1. Problem Description

**Figure 1:**
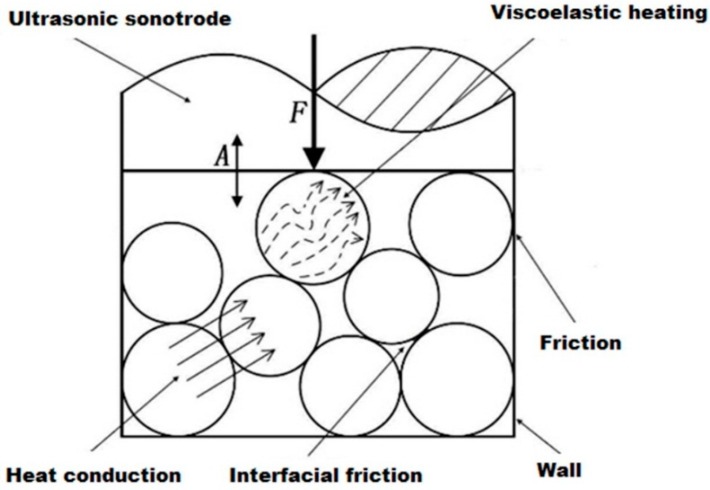
Schematic diagram of heating effects during ultrasonic polymer plasticizing.

In ultrasonic polymer plasticizing, polymer in granulate form is compacted under a certain pressure and plasticized by the sonotrode under ultrasonic vibration as shown in Figure 1. The interfacial friction heating involves surface friction among granulates and between granulates and the wall. The viscoelastic heating is related to the deformation of polymer granulates under ultrasonic vibration. The combined action of the interfacial friction heating and viscoelastic heating leads to a polymer phase change from glassy state to rubbery state. After that, a polymer melt with good flowability could be acquired due to the ultrasonic cavitation and acoustic streaming effect. In our previous work [5], the characteristics and mechanisms of the interfacial friction heating has been investigated. It was found that the interfacial friction heating occurs and lasts only until the interfaces disappear, meaning that it acts only at the initial stage of the ultrasonic plasticizing process. After that, the volumetric viscoelastic heating is dominant. The scope of this research is to promote understanding on the viscoelastic heating mechanism in ultrasonic plasticizing.

### 2.2. Theoretical Modeling

#### 2.2.1. Physical model

**Figure 2:**
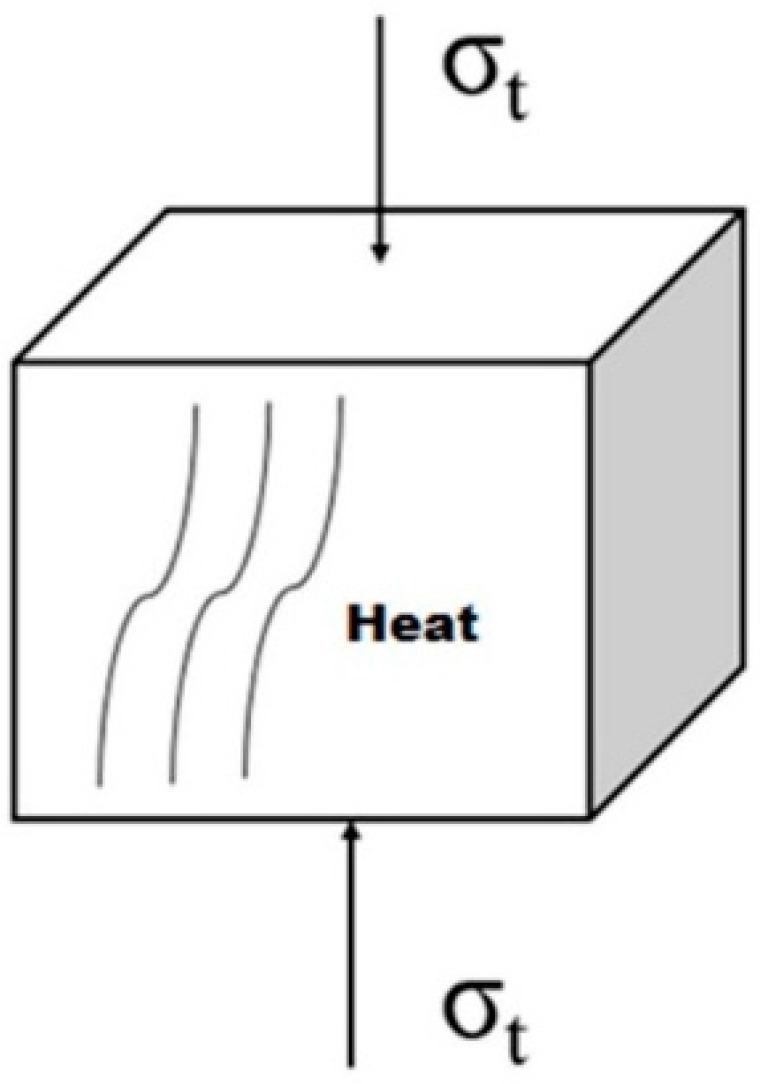
Simplified loading conditions of a micro-unit cell in a polymer granulate.

**Figure 3:**
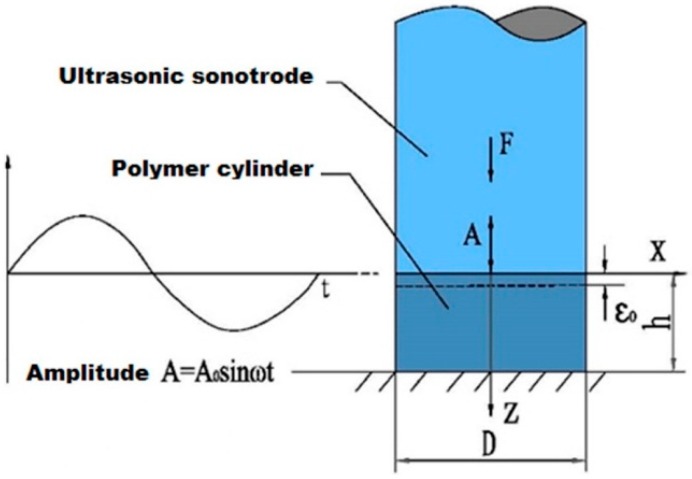
Simplified viscoelastic heating model in ultrasonic plasticizing.

In ultrasonic polymer plasticizing, the stress field in the polymer granulates is usually uneven and complex. To simplify the problem, the loading conditions of a micro unit cell in a polymer granulate is simplified as shown in Figure 2. It is assumed that the micro-unit cell is loaded with ideal uniaxial normal stress σ(t), which is a sine function with the same frequency as the ultrasonic vibration. After being compacted by the plasticizing pressure and heated by the initial interfacial friction, the polymer granulates are assumed to form a homogeneous bulk cylinder without having internal imperfections to facilitate the research. The polymer bulk cylinder has a diameter of 10 mm and a height of 5 mm and is cyclically loaded by the ultrasonic sonotrode as shown in Figure 3. The cyclic load is assumed to have the same amplitude and frequency as the ultrasonic vibration. As a result, the polymer cylinder is supposed to be heated due to the viscoelastic nature of the polymer material.

#### 2.2.2. Mathematical Modeling

**Figure 4:**
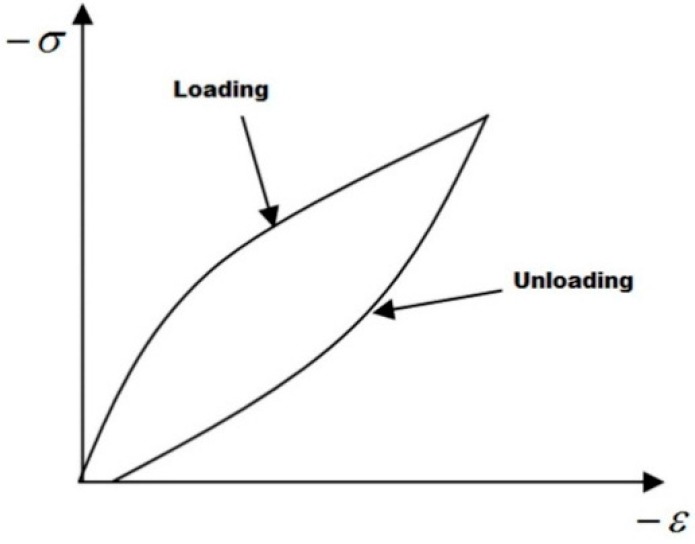
Typical stress-strain curve of polymer material in a vibration cycle.

Figure 4 illustrates a typical stress–strain curve of polymer material in a vibration cycle. A hysteresis loop is formed due to the non-coincidence of the loading and unloading curves. The area of the hysteresis loop is the work done by the ultrasonic sonotrode against the internal friction of the macromolecular segments in a single vibration cycle. Hence, the heat generation of the micro unit cell per unit of time is given by:

(1) $$Q=f\oint \sigma (t)d\varepsilon (t)=f\sigma_{0}\varepsilon_{0}\omega \int_{0}^{2\pi /\omega }\sin\omega t\cos\left(\omega t-\delta \right)dt=f\pi \sigma_{0}\varepsilon_{0}\sin\delta$$

where, f is the vibration frequency, $\sigma_{0}$ and $\varepsilon_{0}$ are, respectively, the amplitude of the stress and strain, δ is the lag angle of the strain, and ω is the angular frequency of the sonotrode.

By expressing the stress and strain in complex number form, the complex modulus of polymer can be simplified as:

(2) $$E^{*}=\left(\frac{\sigma_{0}}{\varepsilon_{0}}\right)\left(\cos\delta +i\sin\delta \right)=E^{\prime }+iE^{\prime \prime }$$

with $E^{\prime }$ is the storage modulus and $E^{\prime \prime }$ is the loss modulus.

By substituting $E^{\prime \prime }=\sigma_{0}\sin\delta /\varepsilon_{0}$ in Equation (1) the heat generation rate Q can be reformulated as:

(3) $$Q=f\pi \varepsilon_{0}{}^{2}E^{\prime \prime }$$

According to Equation (3), it can be deduced that the viscoelastic heating is related to the ultrasonic frequency, the amplitude and the loss modulus of the polymer material.

Considering the attenuation of the ultrasonic amplitude in the propagation direction, the solution of the ultrasonic wave function in the polymer bulk cylinder (see Figure 3) is given by [17,18]:

(4) $$U\left(z,t\right)=U_{0}e^{-\alpha z}\times \exp\{i\omega \left(t-\frac{z}{c}\right)\}$$

where U is the vibration amplitude, c is the propagation speed of sound, z is the distance to sonotrode, and α is the attenuation factor which is given by:

(5) $$\alpha =\frac{\omega \tan\delta }{c}z=\omega z\frac{\omega E^{\prime \prime }}{E^{\prime }}\sqrt{\frac{\rho }{E^{\prime }}}$$

Combining Equations (3)–(5) gives the heat generation rate considering the ultrasonic attenuation:

(6) $$Q(z)=f\varepsilon_{0}{}^{2}\exp\{\frac{-2E^{\prime \prime }\omega z}{E^{\prime }\sqrt{\frac{\rho }{E^{\prime }}}}\}E^{\prime \prime }$$

The modulus of polymers can be influenced by both temperature and loading frequency. For the polymers under low frequency loading, the loss modulus can be measured by dynamic mechanical analysis. For the polymer under high-frequency loading such as the ultrasonic vibration, however, the loss modulus can only be acquired by deducing with the Time-Temperature-Superposition method [19,20,21]. To this end, the complex modulus as a function of temperature and frequency is described by the generalized Maxell model:

(7) $$E^{*}=\sum_{j=1}^{n}\frac{E_{j}\omega^{2}\tau_{j}{}^{2}}{1+\omega^{2}\tau_{j}{}^{2}}+i\sum_{j=1}^{n}\frac{E_{j}\omega \tau_{j}}{1+\omega^{2}\tau_{j}{}^{2}}$$

where j is the generalized Maxwell order number, $E_{j}$ and $\tau_{j}$ are, respectively, the static relaxation modulus and the relaxation time of corresponding Maxwell unit, and ω is the angular frequency of external loading.

The relaxation time is calculated using Arrhenius [11] and semi-empirical WLF equation [15,16]:

(8) $$\begin{array}{l} \tau_{j}(T)=10^{\frac{H}{2.303R}\left(\frac{1}{T}-\frac{1}{T_{s}}\right)}\times \tau_{j}\left(T_{s}\right)\;T\leq T_{g}\\ \tau_{j}(T)=10^{\frac{-C_{1}\left(T-T_{s}\right)}{C_{2}+T-T_{s}}}\times \tau_{j}\left(T_{s}\right)\;T_{g}<T\leq T_{g}+60 \end{array}$$

with $\tau_{T}$ and $\tau_{s}$ being, respectively, the relaxation time at an arbitrary temperature T and the reference temperature $T_{s}$, H is the activation energy, R is the molar gas constant, $C_{1}$ and $C_{2}$ are constants.

By Equations (6)–(8) the viscoelastic heating rate as a function of frequency and temperature is given by:

(9) $$\begin{array}{ll} Q\left(T,\omega ,z\right)=f\pi \varepsilon_{0}{}^{2}\exp\{\frac{-2E^{\prime \prime }\omega z}{E^{\prime }\sqrt{\frac{\rho }{E^{\prime }}}}\}\sum_{j=1}^{n}\frac{E_{j}\omega 10^{\frac{H}{2.303R}\left(\frac{1}{T}-\frac{1}{T_{s}}\right)}\times \tau_{j}\left(T_{0}\right)}{1+\omega^{2}10^{\frac{2H}{2.303R}\left(\frac{1}{T}-\frac{1}{T_{s}}\right)}\times \tau_{j}{}^{2}{\left(T_{0}\right)}^{}} & T\leq T_{g}\\ Q\left(T,\omega ,z\right)=f\pi \varepsilon_{0}{}^{2}\exp\{\frac{-2E^{\prime \prime }\omega z}{E^{\prime }\sqrt{\frac{\rho }{E^{\prime }}}}\}\sum_{j=1}^{n}\frac{E_{j}\omega 10^{\frac{-C_{1}\left(T-T_{s}\right)}{C_{2}+T-T_{s}}}•\tau_{j}\left(T_{0}\right)}{1+\omega^{2}10^{\frac{-2C_{1}\left(T-T_{s}\right)}{C_{2}+T-T_{s}}}•\tau_{j}{}^{2}\left(T_{0}\right)} & T_{g}<T\leq T_{g}+60 \end{array}$$

#### 2.2.3. Numerical Simulation

The governing differential equation to be solved is:

(10) $$\rho C(T)\frac{\partial T}{\partial t}=k_{x}\frac{\partial^{2}T}{\partial x^{2}}+k_{y}\frac{\partial^{2}T}{\partial y^{2}}+k_{z}\frac{\partial^{2}T}{\partial z^{2}}+Q(T,z,\omega )$$

where ρ is density, C(T) is specific heat capacity as a function of temperature T, t is time, $k_{x}$, $k_{y}$, and $k_{z}$ are, respectively, heat conductivity in coordinate directions. Equation (10) can be numerically solved by the heat analysis module in commercial FEM software ANSYS. To this end, the polymer bulk cylinder (see Figure 3) was modeled in ANSYS and meshed by SOLID90 element. The viscoelastic heating rate under certain frequency is defined as a function of the node temperature T and the node coordinate z. By applying suitable assumptions, material properties, and initial and boundary conditions, the numerical solution of the temperature T as a function of time in Equation (10) was obtained by time dependent heat analysis.

- Assumptions
  (1) The heat exchange between the model and the environment is natural convection.
  (2) The contact between the sonotrode and the polymer cylinder stays unchanged during vibration due to the constant plasticizing pressure.
  (3) The density of the polymer bulk cylinder is assumed to be constant during viscoelastic heating to simplify the numerical simulation.
  (4) The heat generated by the sonotrode during ultrasonic plasticizing is neglected to focus on the polymer internal heat generation by viscoelastic heating.
  (5) The possible energy leak from the sensor insertion is neglected because the ultrasonic plasticizing is a transient process which usually lasts only a few seconds.
- Material properties
  (1) The material applied in the model is amorphous poly(methyl methacrylate) (PMMA) which has a glass transition temperature of 105 °C and a viscos flow temperature of 160 °C.
  (2) The relaxation modulus and relaxation time of the generalized Maxwell model at a reference temperature of 105 °C is defined according to [12].
  (3) The activation energy H and the constants $C_{1}$ and $C_{2}$ in Equation (8) are defined as 335 KJ/mol, 17.6, and 65.5, respectively, according to [15].
- Initial conditions
  (1) For the study on the influence of the initial temperature of polymer on the viscoelastic heating, the initial temperature of the polymer was defined as 30, 96, and 100 °C, respectively.
  (2) For the study on the influence of the ultrasonic amplitude and frequency on the viscoelastic heating, the initial temperature was defined as 100 °C.
- Boundary conditions
  (1) The environment temperature is 25 °C.
  (2) There is thermal radiation to environment during viscoelastic heating which was considered by applying the convection and radiation heat transfer coefficient which is defined as 16 W/m^2^·°C.
  (3) For the study on the influence of the initial temperature of polymer on the viscoelastic heating, the vibration frequency and amplitude at the end of the sonotrode were defined as 20 kHz and 40 μm, respectively.
  (4) For the study on the influence of ultrasonic frequency on the viscoelastic heating, a vibration frequency sweep from 15 to 30 kHz with a step of 5 kHz and an amplitude of 40 μm were set up for the end of the sonotrode.
  (5) For the study on the influence of ultrasonic amplitude on the viscoelastic heating, a vibration amplitude sweep from 20 to 50 μm with a step of 10 μm and a vibration frequency of 20 kHz were set up for the sonotrode.

## 3. Experimentation

### 3.1. Materials

A polymethyl methacrylate (PMMA) bar with a diameter of 10 mm was purchased for the viscoelastic heating experiment. The material properties are shown in Table 1.

### 3.2. Ultrasonic Plasticization System

Table 2 indicates the main technical data of the self-developed ultrasonic plasticization system which was used for the viscoelastic heating experiment. It should be clarified that the frequency and the amplitude ranges in numerical simulation may out of what are shown in Table 2. This can be related to the resonance characteristics of the ultrasonic vibration. There are usually only one resonance frequency for a certain ultrasonic vibration system. The experimentation could be too expensive if the frequency and amplitude ranges in the numerical simulation were all covered. Therefore, the frequency and amplitude were chosen according to the numerical simulation results, related open literature [5,6,7], and the research budget.

### 3.3. Methodology

**Figure 5:**
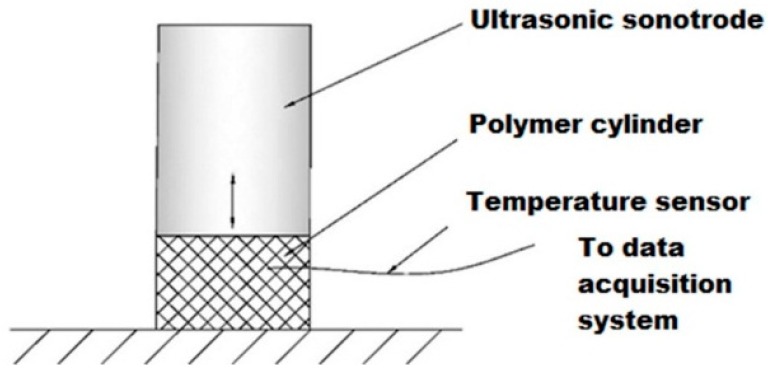
Schematic diagram of viscoelastic heat temperature acquisition method.

To focus on the volumetric viscoelastic heating effect, the influence of the interfacial friction heating should be excluded to avoid uneven plasticization. In addition, an ultrasonic amplitude range from 0–30 um, which is narrower than the one applied in the numerical modeling, was chosen to avoid polymer degradation [5,6,7]. Instead of polymer granulates, a long PMMA bar which was cut into several bulk cylinders with the same size as indicated in Section 2.2.1 was used in the experimentation. Each bulk cylinder was used only for one experiment. As illustrated in Figure 5, a micro-temperature sensor with a probe diameter of 0.1 mm and a response time of 50 ms (Omega, 5TC-TT-K-40-36, Stamford, CT, USA) was inserted into the PMMA bulk cylinder to measure the internal temperature rise under ultrasonic vibration. As the overall temperature of the sample is not evenly distributed due to the attenuation of the ultrasonic vibration, the temperature sensors was tailored to the same embedded location among the bulk cylinders. Specifically, the embedded location of the temperature sensor was 2 mm below the upper surface of the bulk cylinder. The measurements were conducted under various conditions referring to the initial and boundary conditions in Section 2.2.3 and compared with the calculated results from numerical modeling.

## 4 Results and Discussion

### 4.1. Influence of the Initial Temperature of Polymer

**Figure 6:**
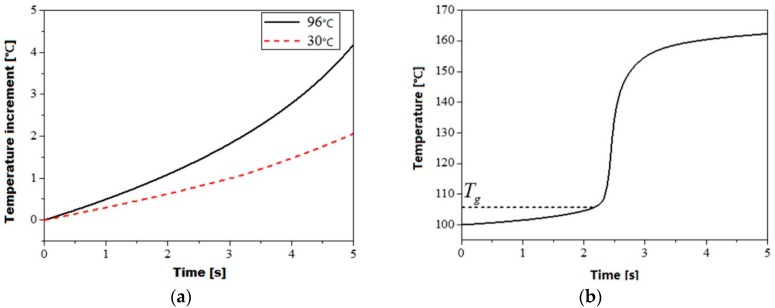
Simulated viscoelastic heating curve of PMMA at various initial temperatures, (**a**) Initial temperature of 30 and 96 °C; (**b**) Initial temperature of 100 °C.

The numerical simulation results indicate that the viscoelastic heating rate can be significantly influenced by the initial temperature of polymer. It is quite obvious that there is a distinct transition of the viscoelastic heating rate below and above the glass transition temperature as shown in Figure 6a,b. For an initial temperature below the glass transition temperature, there is only a slight increment of viscoelastic heating rate with increasing initial temperature as shown in Figure 6a. When the initial temperature of polymer is increased from 30 to 96 °C, the average heating rate has only a slight increment of 0.4 °C/s in 5 s. This can be attributed to the very low loss modulus of the glassy state of the polymer, further lead to very low viscoelastic heating rate according to Equation (3) as illustrated in Section 2.2.2.

When the initial temperature of polymer is further increased to 100 °C, there is still no significant improvement in the viscoelastic heating rate. The average heating rate from 100 to 105 °C is about 2.4 °C/s. With the temperature increases up to 105 °C, a steep increase of the average heating rate up to 64.8 °C/s was determined from 105 to 150 °C. This can be ascribed to the significantly increased loss modulus during the glass transition of PMMA. The average heating rate above 150 °C was found to be reduced to a comparative level that is nearly the same as the one below glass transition temperature. This means that the viscoelastic heating is only highly active in the temperature range between 105 and 150 °C, corresponding to the glass transition range of PMMA.

The variation of the loss modulus can be further explained by the movement of the polymer chain under different material states. Specifically, the polymer cylinder will undergo glassy, viscoelastic and rubbery states in ultrasonic plasticizing. For the glassy state PMMA the deformation of the polymer chain is mainly limited as the changes in the length and the angle of the intramolecular covalent bond and the lateral motion. The material shows typical elastic behavior with inconspicuous stress-strain hysteresis. Hence, the amount of energy dissipated in the form of heat is negligible and the loss modulus is marginal as well. For the viscoelastic state PMMA the macromolecular segments starts to move at temperatures above glass transition temperature and undergo the transformation from the rigid glassy state to the soft rubbery state. The amount of energy dissipated in form of heat can be greatly increased by the significant intermolecular forces, leading to a significantly increased loss modulus. As PMMA enters a rubbery state the macromolecular segments have more freedom of movement, leading to a decreased amount of energy dissipation and loss modulus.

### 4.2. Influence of the Ultrasonic Frequency

**Figure 7:**
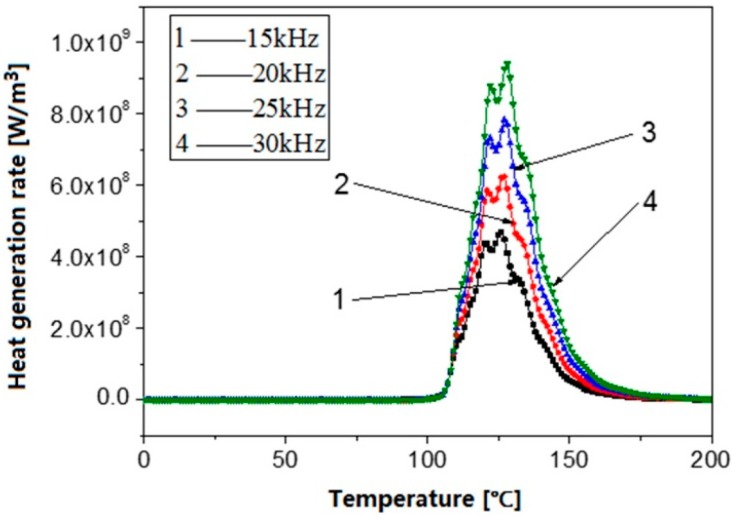
Influence of ultrasonic frequency on the viscoelastic heat generation rate.

**Figure 8:**
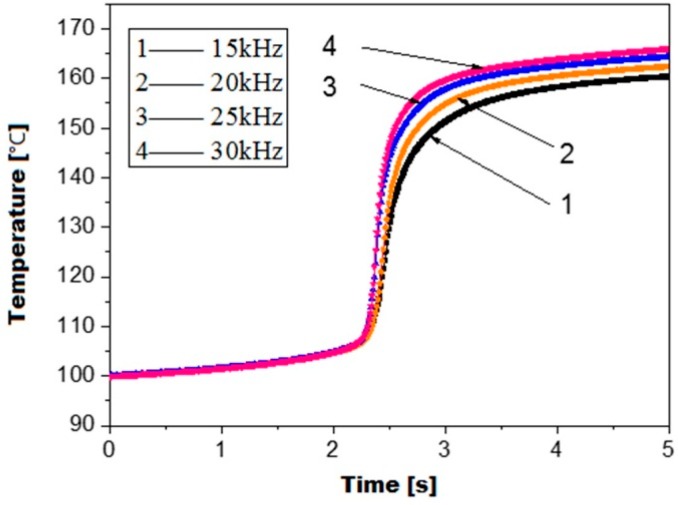
Viscoelastic heating curve of PMMA at various ultrasonic frequencies.

Figure 7 shows the influence of the ultrasonic frequency on the viscoelastic heating rate. It can be observed that the viscoelastic heating rate seems to start and end at zero and is only remarkable at the glass transition range between 105 and 150 °C regardless of the ultrasonic frequency. It should be noted that the viscoelastic heating rate seems to start and end at zero is because of the huge difference in the order of magnitude of viscoelastic heating rate. The order of magnitude out of the glass transition range is too small to compare with the one in the glass transition range. For all ultrasonic frequencies, the viscoelastic heating rate reaches a maximum at about 125 °C, corresponding to the maximum loss modulus at this point during glass transition. The maximum viscoelastic heating rate increases with increasing ultrasonic frequency. The maximum viscoelastic heating rate at 30 kHz ultrasonic frequency is about two times of the one at 15 kHz ultrasonic frequency. However, when observing the temperature curves in Figure 8, it can be found that the ultrasonic frequency has only a limited influence on the polymer temperature rise. The polymer temperature has only a steep increase in the glass transition range between 105 and 150 °C for all ultrasonic frequencies. After plasticizing with an ultrasonic frequency of 30 Hz for 5 s the polymer temperature is only 5.6 °C higher than the one at 15 Hz. Therefore, there is not much meaning to increase the ultrasonic frequency for an increased efficiency of ultrasonic plasticizing.

### 4.3. Influence of the Ultrasonic Amplitude

**Figure 9:**
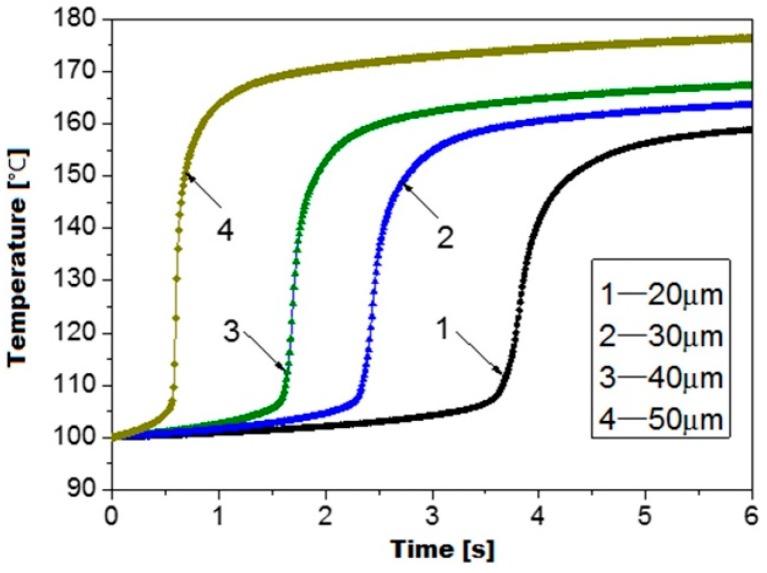
Viscoelastic heating curve of PMMA at various ultrasonic amplitudes.

Figure 9 illustrates the viscoelastic heating curve under various ultrasonic amplitudes. It is as expected that the viscoelastic heating rate can be significantly influenced by the ultrasonic amplitude. Since the viscoelastic heating rate is proportional to the square of the strain amplitude as indicated in Section 2.2.2. The temperature curves illustrate the same trend but with a huge difference in viscoelastic heating rate. It can be determined that the viscoelastic heating rate at 50 μm ultrasonic amplitude is nearly eight times of the one at 20 μm ultrasonic amplitude. Hence, the efficiency of ultrasonic plasticizing can be improved by increasing the ultrasonic amplitude. However, it has been reported by Sacristán *et al.* [6] that too high an ultrasonic amplitude may lead to a decrease of the average molar mass of the polymer. Therefore, a lower ultrasonic amplitude range from 0 to 30 μm was chosen for the experimentation.

**Figure 10:**
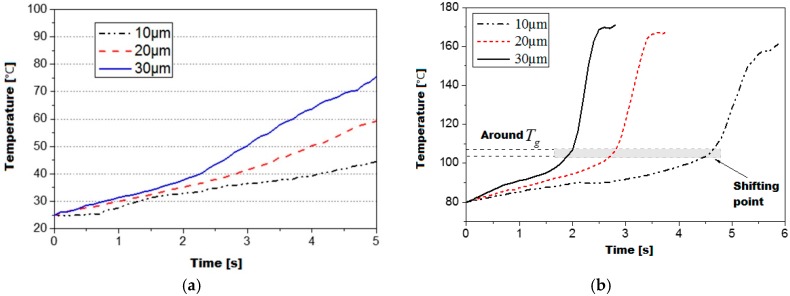
Measured viscoelastic heating curve of PMMA at various initial temperatures, (**a**) initial temperature of 25 °C; and (**b**) initial temperature of 80 °C.

The experimental results further confirms the numerical simulation as shown in Figure 10a,b. The temperature curves measured in the PMMA cylinder show nearly the same trend as the simulated results in the case of both below and above the glass transition temperature. For the PMMA cylinder with an initial temperature of 25 °C, the measured temperature increment is 19.5, 34.4, and 50.6 °C after ultrasonic plasticizing for 5 s, respectively, for the ultrasonic amplitudes of 10 um, 20 um and 30 um as shown in Figure 10a. It was determined that the viscoelastic heating rate at the ultrasonic amplitude of 30 um is about 2.5 times higher than the one at the ultrasonic amplitude of 10 um. For an increased initial temperature of PMMA cylinder, there is no significant change in viscoelastic heating rate until the temperature rises up to 105 °C as shown in Figure 10b. It was determined that the viscoelastic heating rate can be as fast as 125 °C/s at ultrasonic amplitude of 30 um. This is in agreement with results obtained by Tolunay *et al.* [9] They found that the rate of temperature rise can be substantially increased even at places distant from the welding interface when the internal temperature of the polymer reaches up to the temperatures around the glass transition.

It should be noted that although the temperature increase during viscoelastic heating has nearly the same trend, there are still quantitative difference in the rate of viscoelastic heating between experimentation and numerical simulation. For instance, it was determined that the viscoelastic heating rate is about 3.3 °C/s in the range of 100 to 105 °C when the applied ultrasonic amplitude is 30 um in numerical simulation. However, the experimentally-measured viscoelastic heating rate is about 12 °C/s. This may be ascribed to the assumptions made for the simplified numerical simulation model which ignores the heat generation of the sonotrode. In addition, the temperature spectra of loss modulus is unable to accurately obtain under ultrasonic frequency due to the limitation of existing instrument. Hence, the difference in viscoelastic heating rate may be further related to the accuracy of the mathematical modeling of the macromolecular segment movement around glass transition. By the Arrhenius and semi-empirical WLF equations in this work, the influence of temperature on the macromolecular segment movement around glass transition cannot be fully described. Since the theoretical premise of the Arrhenius equation is that the movement units before the glass transition are all chain elements and lateral groups that are smaller than the macromolecular segments. The semi-empirical WLF equation is mainly used to describe the movement characteristics of the macromolecular segments.

## 5. Conclusions

In this work, the viscoelastic heating phenomenon of amorphous polymer in ultrasonic plasticization was studied by both theoretical modeling and experimentation. Considering the attenuation of ultrasonic amplitude in viscoelastic medium, the viscoelastic heating rate was mathematically modelled as a function of the ultrasonic frequency, the square of the strain amplitude and the loss modulus of the polymer. The mathematical model was then applied to the numerical simulation in the commercial FEM software ANSYS. The influence mechanism of several parameters, such as the initial temperature of the polymer, the ultrasonic frequency, and the ultrasonic amplitude were investigated. Both the results from numerical simulation and experimentation indicate that the heat generation rate of viscoelastic heating can be significantly influenced by the initial temperature. The glass transition temperature was found to be a significant shifting point in viscoelastic heating. The heat generation rate is relatively low at the beginning and can have a steep increase after reaching the glass transition temperature. In comparison with the ultrasonic frequency, the ultrasonic amplitude has much greater influence on the heat generation rate. It can be concluded that the viscoelastic heating is the main heat source and predominant in ultrasonic plasticizing of amorphous polymers since the interfacial friction heating lasts only a very short time. For the development of the ultrasonic plasticizing, it would be benefit if the plasticizing chamber could be heated up to the glass transition temperature of amorphous polymers.

## Figures and Tables

**Table 1:** Material properties of PMMA.

Density [Kg/m^3^]	Heat conduction coefficient [W/m·°C]	Specific heat capacity [J/Kg·°C]	Glass transition temperature [°C]	*E*-modulus [GPa]	Poisson’s ratio
1,166	0.18	1,828	105	3.3	0.345

**Table 2:** Technical data of the ultrasonic plasticization system.

Parameter	Power (W)	Frequency (kHz)	Amplitude (μm)	Pressure (Mpa)
Value	0–500	20	0–30	0–30
